# Commercially Viable Hybrid Li-Ion/Metal Batteries with High Energy Density Realized by Symbiotic Anode and Prelithiated Cathode

**DOI:** 10.1007/s40820-022-00899-1

**Published:** 2022-07-22

**Authors:** Kui Lin, Xiaofu Xu, Xianying Qin, Ming Liu, Liang Zhao, Zijin Yang, Qi Liu, Yonghuang Ye, Guohua Chen, Feiyu Kang, Baohua Li

**Affiliations:** 1 Shenzhen Key Laboratory on Power Battery Safety Research and Shenzhen Geim Graphene Center, Tsinghua Shenzhen International Graduate School, Shenzhen, 518055 People’s Republic of China; 2 School of Materials Science and Engineering, Tsinghua University, Beijing, 100084 People’s Republic of China; 3 Contemporary Amperex Technology Co. Ltd., Ningde, 352100 People’s Republic of China; 4 Shenzhen Graphene Innovation Center Co. Ltd., Shenzhen, 518055 People’s Republic of China; 5 College of Materials Science and Engineering, Hunan University, Changsha, 410082 People’s Republic of China; 6 Department of Mechanical Engineering, The Hong Kong Polytechnic University, Hong Kong, 999077 People’s Republic of China

**Keywords:** Hybrid lithium-ion/metal battery, Symbiotic anode, Porous graphite layer, Cathode prelithiation, Lithium oxalate

## Abstract

The energy density of commercial lithium (Li) ion batteries with graphite anode is reaching the limit. It is believed that directly utilizing Li metal as anode without a host could enhance the battery’s energy density to the maximum extent. However, the poor reversibility and infinite volume change of Li metal hinder the realistic implementation of Li metal in battery community. Herein, a commercially viable hybrid Li-ion/metal battery is realized by a coordinated strategy of symbiotic anode and prelithiated cathode. To be specific, a scalable template-removal method is developed to fabricate the porous graphite layer (PGL), which acts as a symbiotic host for Li ion intercalation and subsequent Li metal deposition due to the enhanced lithiophilicity and sufficient ion-conducting pathways. A continuous dissolution-deintercalation mechanism during delithiation process further ensures the elimination of dead Li. As a result, when the excess plating Li reaches 30%, the PGL could deliver an ultrahigh average Coulombic efficiency of 99.5% for 180 cycles with a capacity of 2.48 mAh cm^−2^ in traditional carbonate electrolyte. Meanwhile, an air-stable recrystallized lithium oxalate with high specific capacity (514.3 mAh g^−1^) and moderate operating potential (4.7–5.0 V) is introduced as a sacrificial cathode to compensate the initial loss and provide Li source for subsequent cycles. Based on the prelithiated cathode and initial Li-free symbiotic anode, under a practical-level 3 mAh capacity, the assembled hybrid Li-ion/metal full cell with a *P*/*N* ratio (capacity ratio of LiNi_0.8_Co_0.1_Mn_0.1_O_2_ to graphite) of 1.3 exhibits significantly improved capacity retention after 300 cycles, indicating its great potential for high-energy-density Li batteries.

**Supplementary Information:**

The online version contains supplementary material available at 10.1007/s40820-022-00899-1.

## Highlights

- A symbiotic host of porous graphite layer is designed as hybrid Li anode for Li ion intercalation and subsequent uniform plating.
- An ultrahigh plating reversibility of 99.5% is achieved in the carbonate electrolyte.
- An air-stable cathode prelithiation agent is introduced to provide extra Li source, resulting high energy density of the as-developed practical hybrid Li-ion/metal full cell.

**Supplementary Information:**

The online version contains supplementary material available at 10.1007/s40820-022-00899-1.

## Graphical abstract

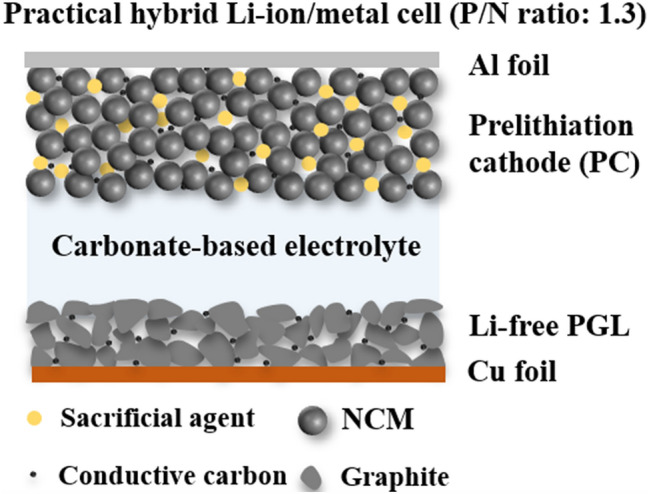

## Introduction

The increasing demand of electrochemical energy storage systems for electric vehicles and grid storage has stimulated intensive scientific and industrial research of high energy density rechargeable batteries beyond concurrent lithium (Li) ion chemistry [1–3]. Among various candidates, anode-free Li metal batteries with lithiated cathode based on the Li plating/stripping mechanism have been regarded as the most promising battery route due to the ultrahigh theoretical capacity (3860 mAh g^−1^) and low reduction potential (− 3.04 V vs standard hydrogen electrode) of Li metal [4–6]. This cell configuration could deliver the maximum possible energy density due to the elimination of active materials (such as graphite, silicon, and tin) as hosts for storing Li ions. This battery system also avoids the handling of highly reactive Li metal during assembling process, which is compatible with the existing production equipment [7]. However, considering that all the limited and active Li ions are initially stored in cathode side, the practical implementation of anode-free Li metal batteries has been heavily impeded by the dendritic growth and poor utilization efficiency of initial plating Li, which is originated from the nonuniform nucleation on planar current collector and the fatal volume variation of Li metal [8, 9].

Over the past few years, several strategies including optimization of electrolyte, modification of current collector, and adjustment of testing parameters have been devoted to improving the electrochemical performance of Li metal batteries by constructing favorable solid electrolyte interphase (SEI) and optimization of delithiation process [10–13]. The development of dual-salt electrolyte, local high-concentration electrolyte or novel electrolyte chemistry could enhance the Coulombic efficiency (CE) of plated Li metal on bare Cu up to 99.5%, which could fulfill the critical requirement of realistic anode-free Li metal batteries [14–18]. However, the volume fluctuation derived from continuous Li plating/stripping on planar current collector should be also took into account, especially in the practical pouch cells [19]. Therefore, it can be concluded that designing porous structures as both hosts and current collectors for accommodating Li deposits and buffering its volumetric change is essential for anode-free Li metal batteries [20, 21]. Diverse metallic scaffolds with different porosity have been introduced to reduce the local current density [22–26]. Unfortunately, Li metal tends to plate at the upper surface of metallic framework due to the much shorter ion-transport pathways, resulting in an unstable electrode/electrolyte interface (as illustrated in Fig. S1). In addition, there will be certain loss in initial CE for porous hosts with larger specific surface area, which is also detrimental for anode-free batteries with finite Li source in cathode. Moreover, the single dissolution delithiation mechanism of plated Li would contribute to the root-stripping behavior, which is adverse for eliminating dead Li. More seriously, the metallic network usually has larger density and delivers chemical inertness for Li ion intercalation, which lower the values for Li metal battery system with desired higher energy density in practical application.

Recently, utilizing lithiated anode materials (graphite or silicon) as hosts for Li metal plating/stripping has been confirmed as an effective strategy to suppress dead Li and enhance overall energy density, assembling as the hybrid Li-ion/metal cells [27, 28]. The lithiated host usually possesses both electronic and ionic conductivity, which could provide sufficient electronic and ionic transfer pathways during metallic plating/stripping process [29, 30]. Meanwhile, the delithiation potential of the lithiated host is slightly higher than that of the metallic Li during stripping, thus ensure a complete stripping of plated Li metal before deintercalation or dealloying process of the lithiated host. In this way, a successive plated Li dissolution and then lithiated host deintercalation mechanism is triggered during delithiation process and the root-stripping behavior can be greatly avoided (as displayed in Fig. S2) [31]. However, in that case, the overtopping surface plated Li and serious volume change still remain unsolved and the improved electrochemical performance is based on personalized electrolyte formula, which is not suitable for practical utilization considering the cost and universality [32, 33]. Therefore, it is highly imperative to develop scalable and cost-effective approaches to construct a symbiotic architecture that composed of active scaffold and abundant accommodation space to achieve staged lithiation from intercalation to deposition, and continuous delithiation containing dissolution and deintercalation mentioned above. In view of the inevitable Li consumption in the electrochemical reactions, especially for the Li-free anode system, prelithiation in the cathode would be a valuable strategy to guarantee the high energy density for the full cells in the whole operating process. It can be believed that combining symbiotic host in anode and prelithiation in cathode would realize the desirable high reversibility and high energy density for the hybrid Li-ion/metal full cells in commercial carbonate-based electrolyte [34].

**Fig. 1:**
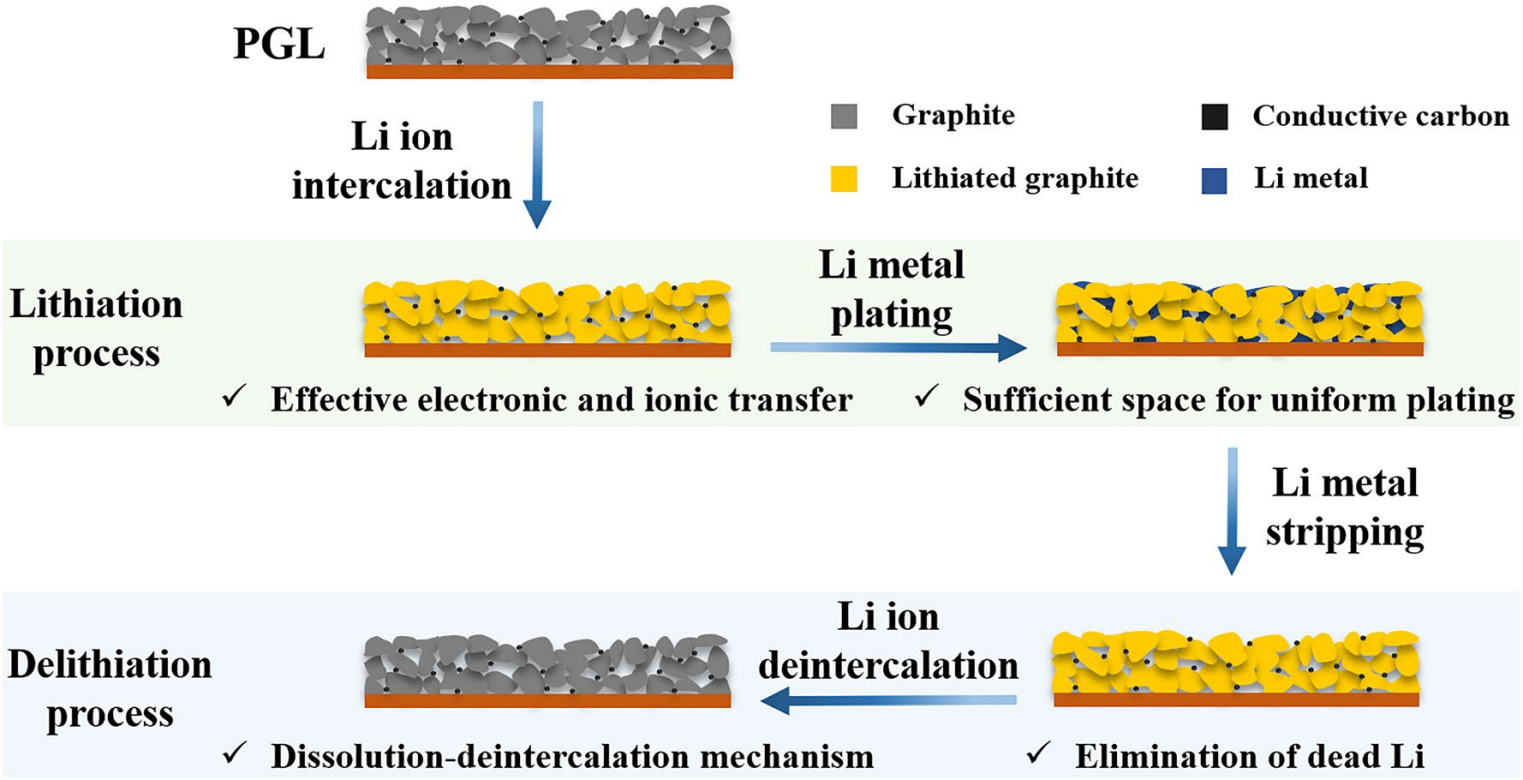
Schematic illustration of lithiation/delithiation behaviors on/from PGL symbiotic anode

In this contribution, we report an industry-available spontaneous template removal method during the electrode drying process for fabricating the porous graphite layer (PGL) on Cu foil, serving as a symbiotic host to accommodate and uniformize Li plating after Li ion intercalation, which is due to the porous architecture and the presence of lithiophilic LiC_6_ with high ion diffusion rate (as shown in Fig. 1). Moreover, the dead Li can be significantly eliminated by the aid of continuous dissolution-deintercalation delithiation mechanism due to the difference of delithiation potential between lithiated graphite and metallic Li. Therefore, equipping with the commercial carbonate-based electrolyte in half cell, the PGL electrode could deliver an ultrahigh CE of 99.5% for 180 cycles with 30% excess plated Li after intercalation (total capacity equals to 2.48 mAh cm^−2^) at 1 mA cm^−2^. Furthermore, in order to construct full cells based on the PGL anode, an air-stable and industrial scalable sacrificial Li salt with high charge specific capacity (514.3 mAh g^−1^), low reversibility (< 10%) and moderate voltage platform (4.7–5.0 V) is directly introduced to the cathode to develop the prelithiated cathode (PC) to compensate the initial capacity loss and provide extra Li source for cycling. As a result, owning to the high utilization efficiency of plated Li metal in PGL, the hybrid Li-ion/metal full cell with a *P*/*N* ratio (the capacity ratio of LiNi_0.8_Co_0.1_Mn_0.1_O_2_ (NCM) to graphite) of 1.3 utilizing PC and Li-free PGL anode could be stably operated for 300 cycles with a capacity retention of 67.2% under a practical capacity depth of 3 mAh, behaving the great potential of these multiple strategies in realistic applications.

## Experimental Section

### Construction of Li-Free Symbiotic Anode

The Li-free symbiotic PGL anode was fabricated by casting the slurry consisting of natural graphite, NH_4_HCO_3_ template, conductive carbon black (CB), polyvinylidene difluoride (PVDF) binder, and N-methyl-2-pyrrolidone (NMP) solvent on the Cu foil followed by drying in an oven at 80 °C for overnight to remove the template. The mass ratio of graphite, NH_4_HCO_3_, CB, and PVDF was 9: 0.7: 0.3: 1 and the as-prepared PGL was punched into a circle pellet with a diameter of 12 mm. For comparison, the conventional graphite layer (CGL) was developed by the same procedures without adding NH_4_HCO_3_ template. The mass loading of graphite on each Cu foil was about 5.3 mg cm^−2^. In addition, the weight ratio of graphite, NH_4_HCO_3_, CB, and PVDF was 9: 0.3: 0.7: 1 for PGL-2 with less porosity, while the ratio was 9: 0.9: 0.1: 1 and 8.5: 1.2: 0.3: 1 for PGL-3 and PGL-4 with more porosity, respectively.

### Preparation of Sacrificial Li Salt and Prelithiated Cathode

The sacrificial cathode agent of recrystallized Li_2_C_2_O_4_ (R-Li_2_C_2_O_4_) was fabricated by a facile recrystallization process from commercial pristine Li_2_C_2_O_4_ (P-Li_2_C_2_O_4_). Typically, the P-Li_2_C_2_O_4_ was dissolved in deionized and the obtained solution was added into the ethanol with a speed of 0.5 mL min^−1^. The mass ratio of P-Li_2_C_2_O_4_, deionized water, and ethanol was 1: 20: 40. The final product R-Li_2_C_2_O_4_ was obtained after vacuum filtration and fully dried. The prelithiated cathode (PC) was prepared by mixing the NCM, R-Li_2_C_2_O_4_, CB, PVDF with a mass ratio of 6.8: 1.2: 1: 1 in NMP solvent and then the slurry was casted into the aluminum (Al) foil followed by drying in a vacuum oven for 12 h. In comparison, the conventional NCM cathode without prelithiation was synthesized by the same procedures with a mass ratio of 8: 1: 1 without introducing the R-Li_2_C_2_O_4_. The average mass loading of NCM in the electrode was about 14.87 mg cm^−2^ (equals to 16.8 mg).

### Material Characterizations

The morphologies of pristine and cycled electrodes were observed by a field emission scanning electron microscope (FE-SEM, HITACHS4800) equipped with an argon (Ar)-filled box, which could protect samples from air. The nitrogen adsorption and desorption isotherms were collected by the Brunauer–Emmett–Teller method at 77 K with Micromeritcs ASAP 2020 analyzer and the corresponding pore size distributions were calculated based on the Barrett-Joyner-Halenda model. X-ray photoelectron spectroscopy (XPS, Physical Electronics PHI5802) measurements were conducted to analyze the surface chemical components of electrode. X-ray diffraction (XRD) patterns of sacrificial cathode agents were tested using the Bruker D8 Advance system using Cu Kα radiation (*λ* = 0.154 nm).

### Electrochemical Measurements

CR2032 coin cells were assembled in an Ar-filled glove box and tested on a Land 2001A battery testing system. The Li plating/stripping reversibility was evaluated in half cell with Li foil as the reference and counter electrode, PGL, CGL or bare Cu foil as the working electrode, Celgard 2400 membrane as the separator, and 1 M LiPF_6_ in fluoroethylene carbonate (FEC)/ethylmethyl carbonate (EMC)/diethyl carbonate (DEC) (volume ratio of 1: 1: 1) as the electrolyte. A fixed amount (50 μL) of the electrolyte was used in each cell and the cells were firstly discharged with a certain amount of Li and then charged with a cut-off voltage of 1.0 V (The current density and capacity of graphite electrode were calculated based on 0.1C = 360 mA g^−1^). Electrochemical impedance spectra (EIS) measurements were conducted utilizing the VMP3 multichannel electrochemical station with the frequency range of 10 mHZ to 100 kHZ by applying the amplitude of 5 mV. The electrochemical behavior of sacrificial cathode agents was characterized by mixing the P-Li_2_C_2_O_4_ or R-Li_2_C_2_O_4_, CB, and PVDF with a weight ratio of 6: 3: 1 in NMP on Al foil. The mass loading was about 0.5 mg cm^−2^ and the current density was 0.5 A g^−1^. For the full cell with Li-free metal anode, the PGL, CGL, or bare Cu foil was directed employed as the anode and the PC or NCM was utilized as the cathode. The electrolyte and separator were the same as that in the half cells and the capacity ratio of NCM cathode to graphite anode (*P*/*N* ratio) was controlled at 1.3. These cells were firstly galvanostatically charged/discharged between 2.5 and 5.0 V at 0.1C for one cycle and then charged/discharged between 2.5 and 4.3 V at 0.1C for one cycle, and finally charged/discharged between 2.5 and 4.3 V at 0.5C for the following cycles (1C = 180 mA g^−1^).

## Results and Discussion

### Preparation and Characterization of PGL Electrode

**Fig. 2:**
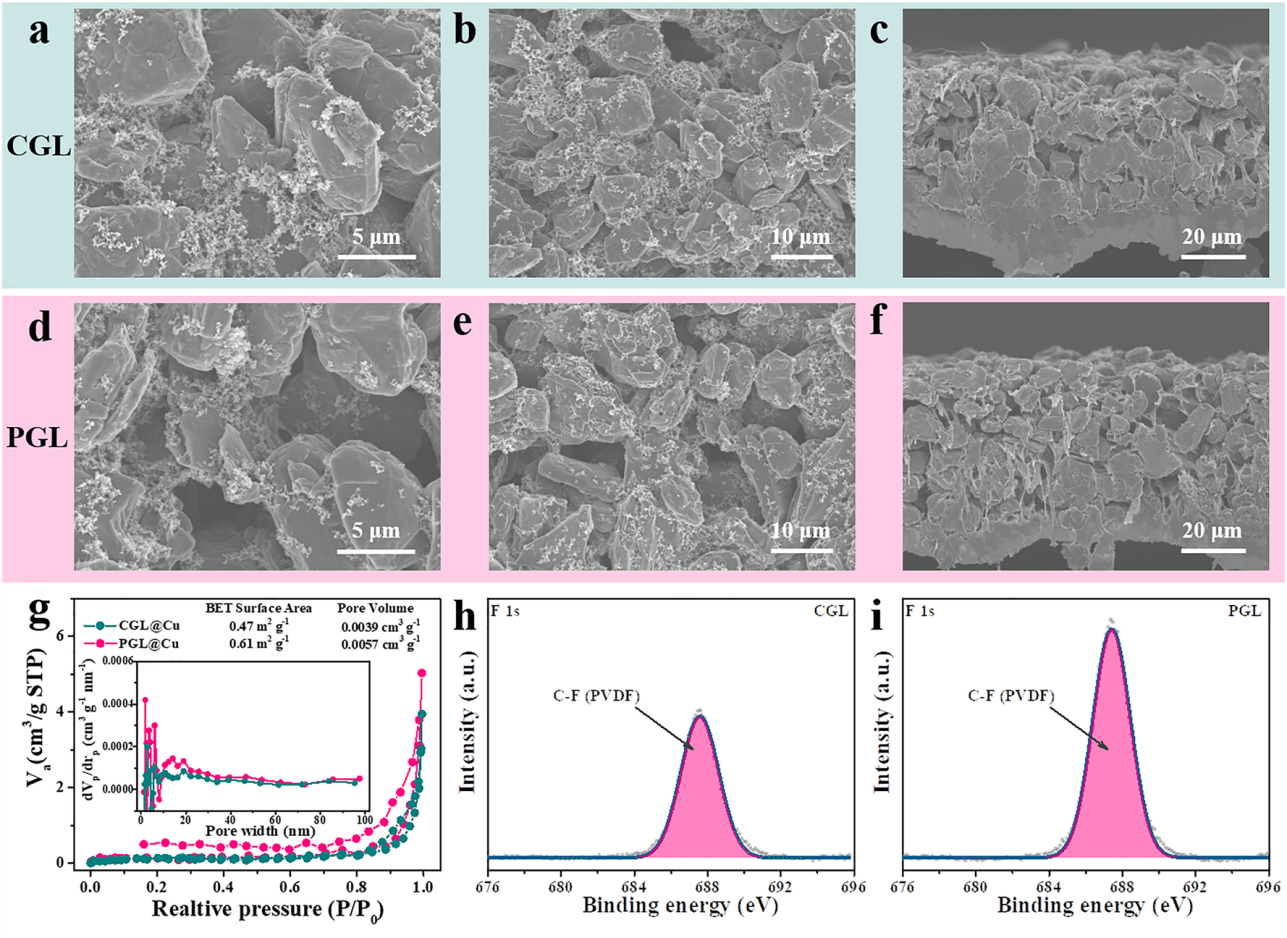
Characterization of CGL and PGL electrodes. **a**, **b** Top-view and **c** cross-view SEM images of CGL. **d**, **e** Top-view and **f** cross-view SEM images of PGL. **g** N_2_ adsorption and desorption isotherms of CGL@Cu and PGL@Cu electrodes (inset: the corresponding pore size distributions). The fine XPS spectra of F 1*s* of **h** CGL and **i** PGL

The micro-structures of CGL and PGL electrodes are characterized by SEM images from top view and cross-view as shown in Fig. 2a–f. The particle size of natural graphite is about 5–10 μm and a few certain voids between natural graphite particles are observed (Fig. 2a, b), which can also be evidenced by the cross-sectional SEM image in Fig. 2c and the thickness of CGL is about 30 μm. The PGL is fabricated by a template removal method by utilizing the NH_4_HCO_3_, which is commercially available and could be decomposed into carbon dioxide, ammonia and water without any deleterious by-products when the temperature reaches 60 °C. Thus, the NH_4_HCO_3_ template can be totally removed spontaneously during the necessary drying process of electrode, which means that no extra process is required. As presented in Fig. 2d–f, besides the voids between particles, there are obvious interconnected pores ranging from 5 to 10 μm uniformly distributed among the graphite particles in the PGL and the thickness of PGL is feasibly tailored at 40 μm by adjusting the thickness of doctor blade, which could ensure an identical graphite mass loading with CGL for the following investigation. In addition, the amount of added NH_4_HCO_3_ template is feasibly controllable and the porosity of PGL can be adjusted according to the realistic demand, which is synthesized and discussed in the following section. The higher porosity of PGL can be further roughly confirmed by the N_2_ adsorption/desorption measurements as shown in Fig. 2g. Taking the contribution proportion of Cu foil into consideration, the specific surface area (SSA) of CGL@Cu electrode is calculated to be 0.47 m^2^ g^−1^, while the SSA of PGL@Cu electrode increases to 0.61 m^2^ g^−1^, which is beneficial to decrease surface local current density and provide abundant nucleation sites for Li plating. In addition, the total pore volume of PGL@Cu electrode (0.0057 cm^3^ g^−1^) is also larger than that of CGL@Cu electrode (0.0039 cm^3^ g^−1^) and the inset pore size distributions manifest that more hierarchical pores are generated in PGL electrode, which would facilitate the access of electrolyte and offer sufficient space for Li storing. XPS results of F 1*s* spectra displayed in Fig. 2h, i indicate that there is more PVDF binder exposed in PGL due to its porous structure, which can be further confirmed by the C 1*s* spectra in Fig. S3. The higher F content from PVDF may enhance the capability of electrode in buffering mechanical stress during volume expansion and also promote the formation of rigid F-rich SEI for favorable plating and efficient stripping [35–37].

### Li Plating/Stripping Reversibility on PGL Electrode

**Fig. 3:**
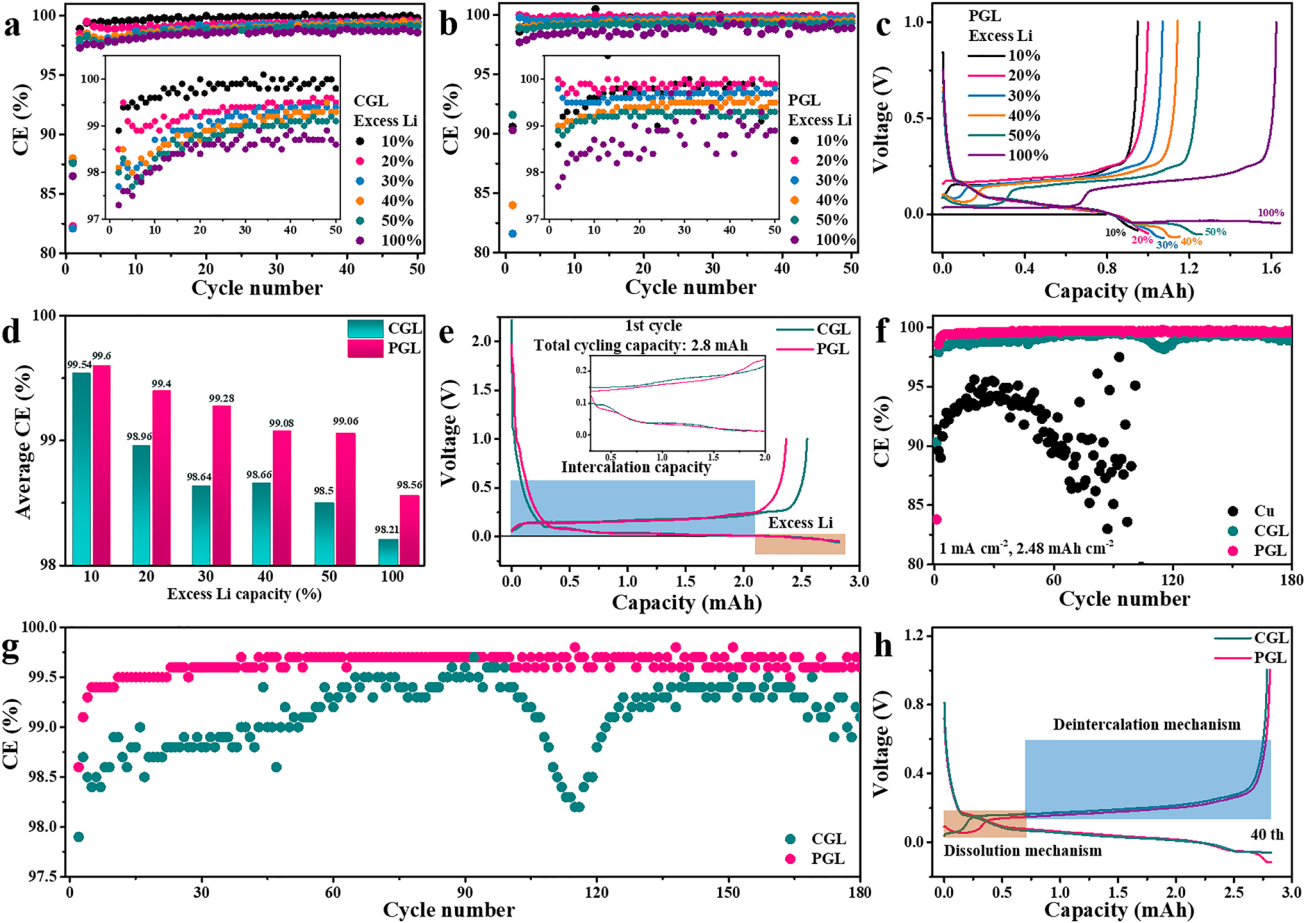
The evaluation of plating/stripping reversibility with various excess Li on/from CGL and PGL in half cells. CE comparisons of **a** CGL and **b** PGL when plating with various amounts of excess Li of 10%, 20%, 30%, 40%, 50%, and 100% under a plating/stripping rate of 1 mA cm^−2^ (inset: magnified CE values, respectively). **c** The corresponding voltage profiles of PGL under various amount of excess Li. **d** Average CE comparisons of CGL and PGL under various excess Li percentage for 50 cycles. **e** Typical initial discharge/charge curves of CGL and PGL plating with 30% excess Li under a realistic high capacity of 2.8 mAh (equals to 2.48 mAh cm^−2^) at 1 mA cm^−2^ (inset: magnified voltage profiles). **f** CE stabilities upon repeated cycling of bare Cu, CGL, and PGL at 1 mA cm^−2^ with a capacity of 2.48 mAh cm^−2^. **g** Magnified CE values of CGL and PGL. **h** The 40th cycle voltage profiles of CGL and PGL

The reversibility of Li plating/stripping on/from CGL or PGL electrode is investigated by the CE measurements in half cells by assembling Li foil as the counter and reference electrode. It is noted that the lithiation process contains the intercalation of Li ion and the deposition of Li metal, while the delithiation process is comprised of dissolution of Li metal and deintercalation of Li ion. During the lithiation process, Li metal starts to plate after the graphite is fully intercalated, which can be called the excess Li plating behavior and the amount of excess plated Li can be expressed as the capacity ratio of plated Li to intercalated Li. Here, the definition of CE in half cell is the ratio of sum of stripped and deintercalated Li to the sum of plated and intercalated Li. The Li plating behavior on graphite is frequently regarded as a serious concern in Li-ion battery and is highly correlated with the amount and position of plated Li. We firstly study the influence of excess Li plating after Li ion intercalation on CE fluctuation under a small total lithiation capacity to determine the durable percentage of excess plated Li on CGL and PGL. As depicted in Fig. 3a, the CGL could deliver CE about 99.5% with 10% excess Li due to the existence of some voids for accommodating plated Li, and the CE values decrease below 99% when the amount of excess Li reaches 30% and then become worse along with the increase of excess Li from 40 to 100%. The corresponding voltage curves during charging-discharging process are presented in Fig. S4. In contrast, an ultrahigh CE of 99.5% could still be obtained when there is 30% excess Li on PGL and the average CE is able to maintain above 99% even with 50% excess Li. The corresponding voltage profiles of PGL and average CE values within 50 cycles are summarized in Figs. 3c, d and S5. Based on the aforementioned results, an excess Li amount of 30% is selected for further investigation due to the high CE value of 99.5% in PGL with traditional carbonate electrolyte, which is the prerequisite for making Li metal-based battery practical [38]. The PGL is further assessed under a practical areal capacity depth of 2.48 mAh cm^−2^, which contains the intercalation capacity of 1.91 mAh cm^−2^ (5.3 mg cm^−2^ × 360 mAh g^−1^) and 30% excess Li plating capacity of 0.57 mAh cm^−2^ (1.91 mAh cm^−2^ × 30%). As presented in Fig. 3e, the measured intercalation capacity of PGL is about 1.92 mAh cm^−2^, which includes the capacity from SEI formation and undeveloped capacity of graphite particles. The PGL exhibits the smaller discharge/charge voltage difference compared with the CGL, indicating its better interfacial stability. And the initial CE of PGL is lower than that of CGL, which can be ascribed to its porous feature with higher SSA and pore volume, leading to increased electrolyte infiltration. As depicted in Fig. 3f, g, the PGL could deliver an impressive reversibility with CE above 99.5% within 10 cycles and high average CE of 99.5% for prolonged 180 cycles at 1 mA cm^−2^ with a total high capacity of 2.48 mAh cm^−2^, demonstrating its significant potential in practical full cell applications. In comparison, the CE of CGL is below 99.0% within 100 cycles, indicating the necessity of increasing the porosity of graphite layer to absorb more amount of excess plated Li. Whereas the bare Cu current collector shows much lower CE values (< 95%) and quickly fading after 50 cycles, demonstrating its poor reversibility, which can be ascribed to the uncontrollable Li plating behavior and tremendous dead Li residues. Equally important, during the delithiation process, as revealed in Fig. 3h, due to the deintercalation potential of lithiated graphite is slightly higher than stripping potential of Li metal, the plated Li would first dissolve followed by the deintercalation of LiC_6_, forming a successive delithiation mechanism, which could ensure a complete stripping of plated Li and thus contribute to highly reversibility plating/stripping behaviors. Furthermore, this controllable template-removal method enables us to fabricate PGL with less porosity (named as PGL-2) and more porosity (named as PGL-3 and PGL-4) and tested with various amount of excess Li as presented in Fig. S6. The reversibility of PGL-2 with less porosity becomes obviously worse when the excess plated Li reaches 30%, which can be attributed to the decreased electrode/electrolyte interface stability due to the upper surface plated Li. While the CE values could maintain around high as 99.5% for PGL-3 under 40% excess Li and for PGL-4 under 50% excess Li, respectively. This result manifests that under various circumstances of capacity depth, the high reversibility of PGL can be preserved by matching the amount of excess Li with the structural porosity of graphite layer. In a more practical attempt, the PGL could still deliver decent cycling stability and high CE above 99.5% within initial 20 cycles and around 98% for 100 cycles in the conventional carbonate-based electrolyte without any additives (1 M LiPF_6_ in EC/DEC (volume ratio of 1: 1)), which is also better than that of CGL and greatly outperformed the bare Cu (Fig. S7), indicating its universality in other electrolyte systems.

### Li Plating/Stripping Behaviors on PGL Electrode

**Fig. 4:**
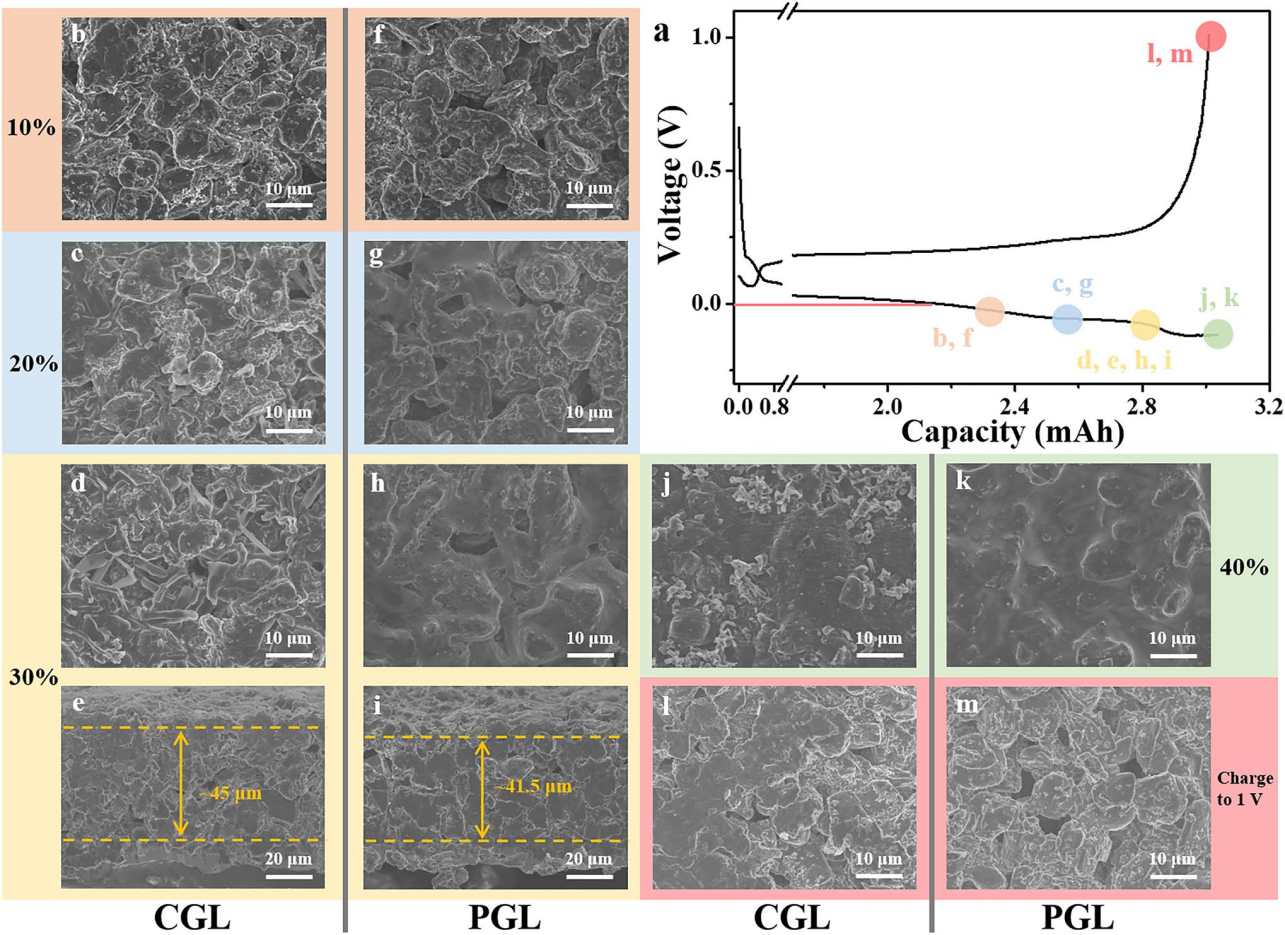
The morphologies of different amounts of excess Li deposited on CGL and PGL and delithiation state of CGL and PGL. **a** Voltage profiles upon plating with various excess Li capacity after intercalating to 0 V and then charging to 1 V. SEM images of CGL after **b** plating 10% excess Li, **c** plating 20% excess Li, **d, e** plating 30% excess Li, **j** plating 40% excess Li, and **l** charging to 1 V. SEM images of PGL after **f** plating 10% excess Li, **g** plating 20% excess Li, **h**, **i** plating 30% excess Li, **k** plating 40% excess Li, and **m** charging to 1 V

To reveal the detailed mechanism behind the significantly enhanced reversibility and cycling stability of PGL electrode, the excess Li plating and stripping behaviors are further studied by ex-situ SEM images. Figure 4a shows the initial discharge/charge curves with marked circles, which represent the different stages during lithiation/delithiation processes. It can be observed that Li is inclined to plate on the top-surface of CGL (Figs. 4b and S8a) and then some protuberances are formed (Figs. 4c and S8b) and gradually evolve into long tubular-shaped and porous Li agglomerations (Figs. 4d and S8c). The corresponding cross-sectional SEM images in Figs. S9a, b and 4e further indicate the unordered Li plating behavior on CGL with the formation of rugged electrode surface and the thickness of electrode is drastically increased to about 45 μm due to the excess porous Li deposits at the interface. In stark contrast, as shown in Fig. 4f, h, Li metal is uniformly plated around the graphite particles and well absorbed into the voids and interconnected pores of PGL, which are gradually cast by the excess deposited Li. From the side perspectives in Figs. S9d, e and 4i, Li metal is well accommodated inside the PGL without growing on the top surface and the overall thickness maintains almost unchanged (about 41.5 μm). When the excess Li amount reaches 40%, the surface of CGL is heavily covered by plated Li with local Li filaments (Fig. 4j) and a notable additional Li layer is clearly observed (Fig. S9c). In comparison, the pores of PGL are fulfilled by the smoothly deposited Li and the graphite particles could still be identified (Fig. 4k). In addition, a flat and clean surface without dendritic humps emerges in the upper section of PGL electrode from the side-view SEM image (Fig. S9f). After reaching the charge cut-off potential, a certain amount of dead Li with apparent height overlapping the graphite particles and voids is left on the top-surface of CGL (Figs. 4l and S10a), meaning its poor cycling reversibility. In contrast, from the top and cross-sectional SEM images (Figs. 4m and S10b), the distinctive pore structure of PGL with empty voids are recovered without the formation of redundant dead Li, which indicates that the deposited Li is able to strip entirely without any residuals. EIS is further measured to assess the interface stability upon repeated cycling with 30% excess Li plating. As shown in Fig. S11, the semicircle in high-frequency reflects the interfacial resistance, including the charge transfer resistance and SEI interfacial resistance at the electrode/electrolyte interface. The interfacial resistance of CGL electrode exhibits an increasing tendency after 30 cycles (Fig. S11a), which could be ascribed to the formation of dead Li byproduct and aggravated SEI film. For comparison, the interfacial resistance of PGL electrode maintains steady without increasing within 30 cycles (Fig. S11b), demonstrating the favorable interfacial stability and elimination of dead Li at the interface.

Based on above case analysis, we can conclude that the porous structure of symbiotic PGL is beneficial for providing sufficient electrode internal space to accommodate excess Li deposition and buffering its infinite volume change. In addition, the intercalation product of graphite is proved to possess high lithiophilicity and favorable ionic conductivity, which could further enhance the uniformity of Li plating and offer abundant ionic transfer pathways [29]. Therefore, the plated Li could be well absorbed within the voids and pores of PGL electrode even the amount of excess Li reaches 40%, and the continuous dissolution-deintercalation delithiation mechanism is also conducive to eliminate the formation of surface Li residues, which synergistically contributes to the promoted interface stability and highly plating/stripping reversibility. For comparison, excess Li being plated on the upper electrode surface of CGL with limited space would lead to deteriorated interfacial stability and unavoidable formation of dead Li, leading to lower Li utilization with shorter cycle life.

### Preparation of PC and Evaluation of Full Cell

**Fig. 5:**
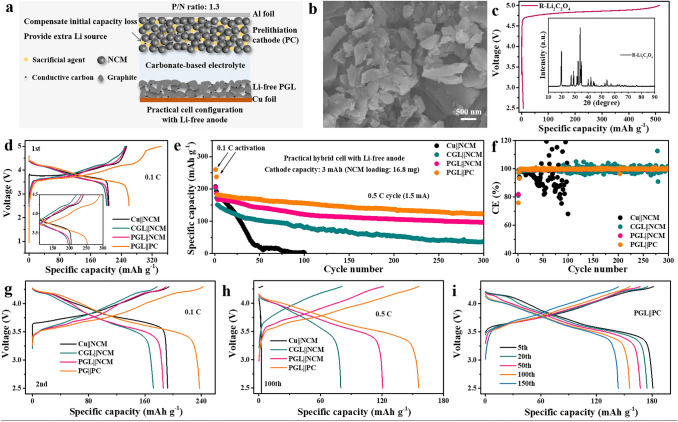
Electrochemical performances of sacrificial cathode agent and full cells. **a** Schematic illustration of practical full cell configuration with initial Li-free PGL anode and PC while using the commercial carbonate-based electrolyte. **b** SEM image of R-Li_2_C_2_O_4_. **c** Charge/discharge curves of R-Li_2_C_2_O_4_ at 500 mA g^−1^ (inset: XRD pattern). **d** Typical initial charge/discharge profiles of four kinds of full cells (Cu||NCM, CGL||NCM, PGL||NCM, and PGL||PC) at 0.1C between 2.5 and 5.0 V (inset: magnified voltage profiles). **e** The cycling performances and **f** corresponding CE values of four kinds of full cells with Li-free anode at 0.5C. **g** The charge/discharge profiles for second cycle of four kinds of full cells at 0.1C between 2.5 and 4.3 V. **h** Charge–discharge profiles for the 100th cycle of four kinds of full cells at 0.5C. **i** Charge–discharge curves upon cycling of the Li-free PGL and PC based hybrid Li-ion/metal battery

The ultrahigh CE and stable lifespan of symbiotic PGL electrode at high capacity depth in half cells with carbonate electrolyte inspire us to evaluate its feasibility in hybrid Li-ion/metal full cell system using high voltage NCM layered oxide as cathode material (the morphology is presented in Fig. S12). The capacity of NCM cathode is controlled as high as 3 mAh, and the corresponding *P*/*N* ratio (capacity ratio of NCM to graphite) is controlled at 1.3 to ensure that the excess plated Li is around 30% of the intercalation capacity in the anode when operating. The full cell configuration is schematically illustrated in Fig. 5a. To minimize the influence of relatively low initial CE of porous scaffold and provide additional Li source for replenishing irreversible Li loss during cycling, it is necessary to prelithiate the cathode with sacrificial Li salt. This has been demonstrated as an effective strategy for fabricating practical Li metal full cells [39–43]. However, the currently reported sacrificial agents, such as Li_2_O and Li_3_N, are air-sensitive and not suitable for actual productions [44, 45]. An ideal cathode prelithiation reagent should possess excellent air stability, high charge specific capacity, low charge/discharge reversibility, reasonable operating voltage range and harmless decomposition products. Here, we introduce lithium oxalate (Li_2_C_2_O_4_) with theoretical charge specific capacity of 525 mAh g^−1^, suitable working voltage of 4.7 V, and low reversibility (< 10%) as an air-stable sacrificial salt [46]. In addition, the decomposition product of Li_2_C_2_O_4_ is Li^+^ and CO_2_, which would not deteriorate the long-term cycling performance since CO_2_ is beneficial for Li deposition and the gas product can also be extracted after formation process to avoid the battery swollen problem [47]. However, the particle size of purchased P-Li_2_C_2_O_4_ is about micron level (Fig. S13a) and the specific capacity is hard to exert fully (working from 4.75 to 5 V with a specific capacity about 402.5 mAh g^−1^, Fig. S13b) due to its low electronic conductivity (10^–10^ S cm^−1^). Therefore, we develop an effective, scalable and low-cost recrystallization method by utilizing the different solubility of Li_2_C_2_O_4_ in ethanol and water to obtain R-Li_2_C_2_O_4_ with significantly reduced particle size of around 200 nm (Fig. 5b), while this process does not affect the phase of Li_2_C_2_O_4_ (seen from the insets of Figs. S13b and 5c). The decomposition voltage of R-Li_2_C_2_O_4_ is decreased to 4.7 V and the specific capacity can be nearly fully utilized with 514.3 mAh g^−1^ when the voltage reaches 5 V. These electrochemical properties indicate that R-Li_2_C_2_O_4_ could serve as an ideal sacrificial cathode agent for fabricating PC. In addition, the amount of sacrificial reagent can be feasibly tailored according to actual requirements and anode materials including silicon or tin. Here, the percentage of added R-Li_2_C_2_O_4_ is 12% based on the overall mass of electrode, which is expected to not only compensate initial CE loss (about 15–20% based on NCM cathode), but also supply additional Li source for replenishing subsequent Li loss. The assembled four kinds of full cell configurations including Cu||NCM, CGL||NCM, PGL||NCM, and PGL||PC are firstly cycled at 0.1C between 2.5 and 5.0 V and the corresponding voltage charge/discharge curves are shown in Fig. 5d. The PGL||PC cell could deliver a charge specific capacity of 324 mAh g^−1^ (based on the mass of NCM) due to the incorporation of R-Li_2_C_2_O_4_. A high reversible specific capacity of 260 mAh g^−1^ is gained for PGL||PC cell, which is much larger than that of three other kinds of full cells, indicating the initial capacity loss that caused by porous scaffold of PGL can be effectively compensated. In addition, the voltage polarizations between the charge and discharge plateaus of PGL||NCM and PGL||PC cells are much smaller than that of CGL||NCM cell, clarifying the construction of stable interface in PGL anode. During the subsequent cycle, the cells are tested between 2.5 and 4.3 V at 0.1C and then at 0.5C for the following long-term cycling and the corresponding voltage curves are summarized as Figs. 5e and S14. The PGL||PC cell still presents the minimal polarization and highest reversible capacity than other cell systems. Figure 5e, f shows the long-term cycling performances and CE values of four kinds of full cells. The Cu||NCM cell suffers a quick capacity fading within 20 cycles, which can be ascribed to the irreversible plating/stripping process originating from uneven plating and unordered stripping on bare Cu current collector with zero-excess Li. While the CGL||NCM cell exhibits discharge capacity smaller than 100 mAh g^−1^ and displays fluctuant CE after 70 cycles, mainly deriving from the unstable electrode/electrolyte interface with excess plated Li. By contrast, the reversible capacity of PGL||NCM cell is largely improved to 96.5 mAh g^−1^ with 57.6% capacity retention (36.3 mAh g^−1^ with 24.1% capacity retention for CGL||NCM), and its CE maintains stable even after 300 cycles. This indicates the importance of constructing porous framework for accommodating excess Li plating within the internal space at electrode level and then enhancing the utilization efficiency of Li metal in the Li-free PGL anode. Further introducing the sacrificial agent of R-Li_2_C_2_O_4_, the PGL||PC cell could be stably operated for 300 cycles with high reversible capacity of 122.3 mAh g^−1^ at a current density of 1.5 mA, which corresponds to a high capacity retention of 67.2%. And the CE ascends to above 99.5% after three cycles and retains around this high value in the long cycling, suggesting that after compensating the initial irreversible capacity loss, the left Li source in sacrificial agent could further provide extra Li to offset continuous loss in long-term cycling. Thus, the capacity fading could be significantly reduced. In addition, the corresponding voltage curves of four kinds of full cells at 100th cycle shown in Fig. 5h also point out the smallest polarizations and highest reversibility of PGL||PC cell among the three styles of full cells. Moreover, the cycling reversibility of PGL||PC cell can also be verified by the charge/discharge curves with little variations from 5th cycle to 150th cycle (Fig. 5i). The fabrication processes and electrochemical performances of this work and representative reported Li metal batteries with initial Li-free or Li-less metal anode are compared and summarized in Table S1. These results indicate that the electrochemical performance of this work is comparable or superior to recently reported works in view of the capacity depth, CE value, current density and long-term cycling lifetime and also demonstrate the commercial feasibility of this hybrid Li-ion/metal full cell configuration. The detailed discussions are illustrated in the Supplementary Information.

## Conclusions

In summary, we develop a feasible, scalable, and industry-available templated-removal method during electrode drying process to construct PGL as the symbiotic anode for hybrid Li-ion/metal battery. The Li ion intercalated PGL with LiC_6_ possesses lithiophilicity and low ion-diffusion energy barrier, which is beneficial for realizing uniform Li deposition. While the abundant internal space in PGL could absorb 30% excess plated Li into the pores/voids within the electrode level, contributing to improved interfacial stability. In addition, the successive dissolution-deintercalation mechanism during delithiation process could eliminate the formation of dead Li. Thus, the reversibility of Li plating/stripping on/from PGL is significantly enhanced to the practical level with an ultrahigh average CE of 99.5% at a capacity depth of 2.48 mAh cm^−2^ for 180 cycles in the carbonate-based electrolyte when the amount of excess Li is 30%. The feasibility of Li-free PGL scaffold in realistic implementation is further demonstrated in the full cell configuration. An air-stable and easily prepared cathode prelithiation agent with high specific capacity and moderate voltage is incorporated to compensate the initial capacity loss of porous scaffold and also provide additional Li source for long-term cycling. Benefiting from the highly reversible process in PGL, the PGL||PC practical full cell with *P*/*N* ratio of 1.3 and zero-excess Li in anode exhibits remarkable CE retention and exceptional cyclability for 300 cycles under the condition of industrially cathode loading. It is believed that these developed facile, closing-to-industry but effective strategies of designing porous symbiotic anode and introducing sacrificial cathode agent to fabricate hybrid Li-ion/metal battery with Li-free anode would present new insights into the development of realistic and high energy density batteries.

## Supplementary Information

Below is the link to the electronic supplementary material.

Supplementary file1 (PDF 884 KB)
