# Supplementary material for: Commercially Viable Hybrid Li-Ion/Metal Batteries with High Energy Density Realized by Symbiotic Anode and Prelithiated Cathode
Source: Nanomicro Lett. 2022 Jul 22;14:149. doi: 10.1007/s40820-022-00899-1 (PMC9307699; doi:10.1007/s40820-022-00899-1)
Supplement: Supplementary file 1 — Supplementary file1 (PDF 884 KB) [file 40820_2022_899_MOESM1_ESM.pdf]

Supporting Information for

Commercially Viable Hybrid Li-Ion/Metal Batteries with High Energy Density Realized by Symbiotic Anode and Prelithiated Cathode

Kui Lin[1, 2, #], Xiaofu Xu[3, #], Xianying Qin[1, 4, *], Ming Liu[1, *], Liang Zhao[1, 2], Zijin Yang[1, 2], Qi Liu[5], Yonghuang Ye[3], Guohua Chen[6], Feiyu Kang[1, 2], Baohua Li[1, *]

[1]Shenzhen Key Laboratory on Power Battery Safety Research and Shenzhen Geim Graphene Center, Tsinghua Shenzhen International Graduate School, Shenzhen 518055, P. R. China

[2]School of Materials Science and Engineering, Tsinghua University, Beijing 100084, P. R. China

[3]Contemporary Amperex Technology Co. Ltd., Ningde 352100, P. R. China

[4]Shenzhen Graphene Innovation Center Co. Ltd., Shenzhen 518055, P. R. China

[5]College of Materials Science and Engineering, Hunan University, Changsha 410082, P. R. China

[6]Department of Mechanical Engineering, The Hong Kong Polytechnic University, Hong Kong 999077, P. R. China

[#] Kui Lin and Xiaofu Xu contributed equally to this work.

*Corresponding authors. E-mail: qinxianying2005@126.com (Xianying Qin), liuming@sz.tsinghua.edu.cn (Ming Liu), libh@mail.sz.tsinghua.edu.cn (Baohua Li)

S1 Supplementary Figures and Tables

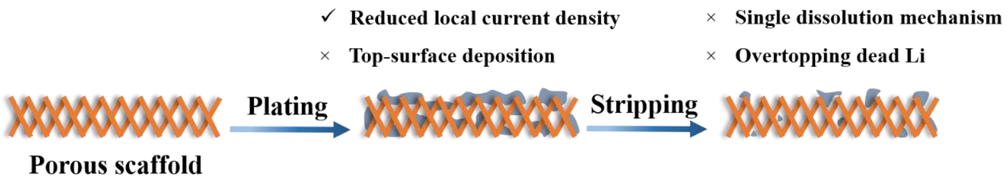

Fig. S1 Schematic illustration of Li plating/stripping behaviors on/from conventional porous scaffold

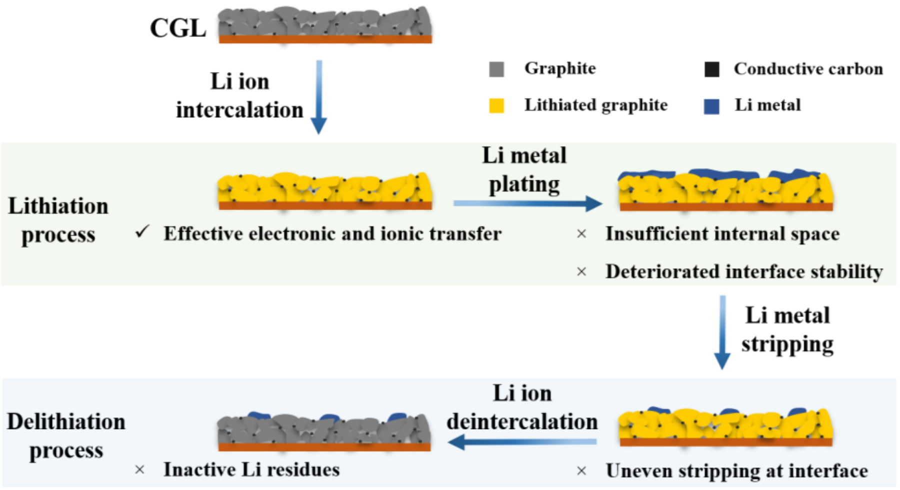

Fig. S2 Schematic illustration of lithiation/delithiation behaviors on/from conventional graphite layer (CGL) electrode

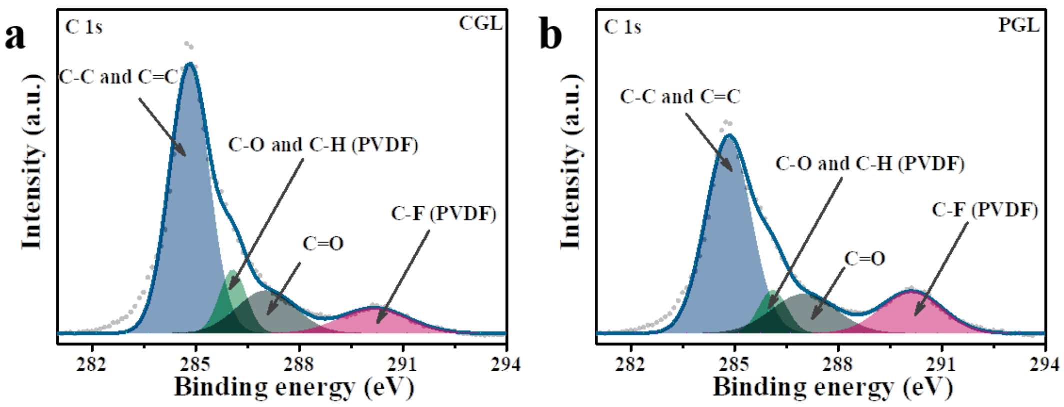

Fig. S3 The fine XPS spectra of C 1s of **a** CGL and **b** porous graphite layer (PGL)

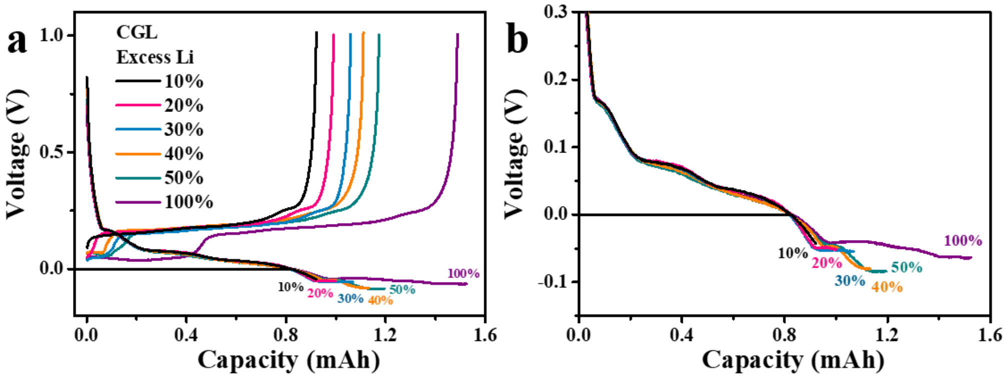

Fig. S4 The voltage profiles of CGL under various amount of excess Li plating

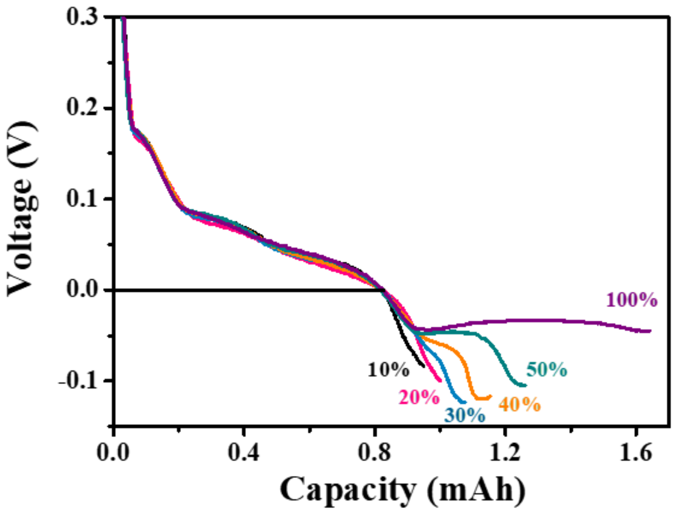

Fig. S5 The magnified voltage profiles of PGL with various amount of excess Li

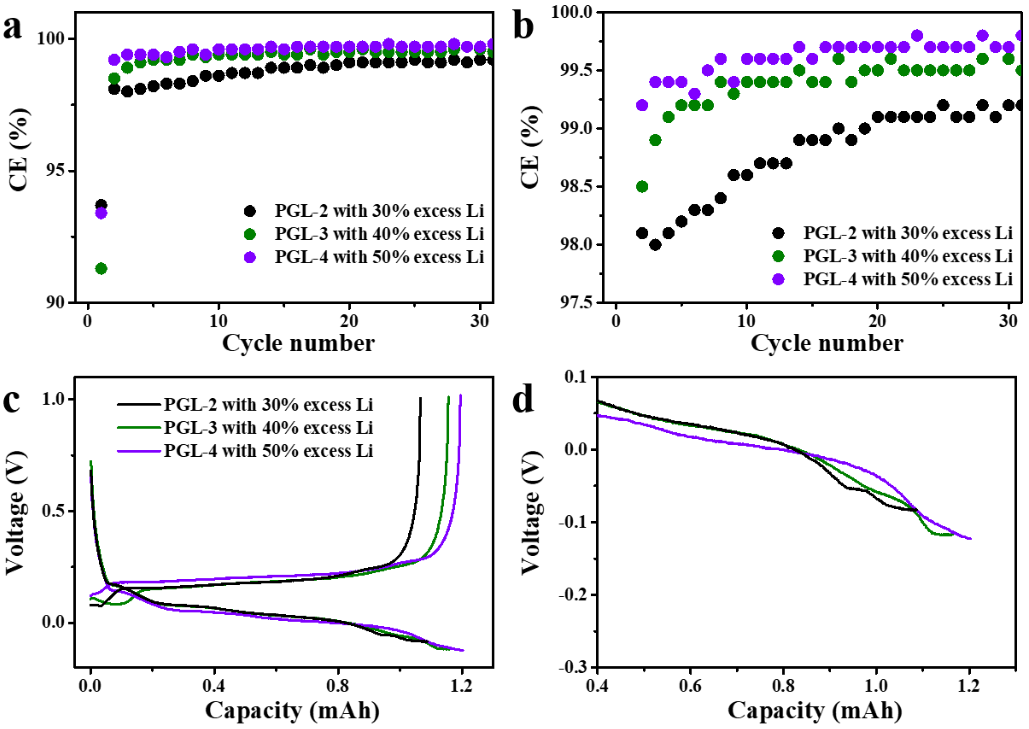

Fig. S6 a CE stabilities and **b** magnified CE values upon repeated cycling of PGL-2 with 30% excess Li, PGL-3 with 40% excess Li, and PGL-4 with 50% excess Li at 1 mA cm^{-2}. **c** The voltage profiles and **d** magnified curves of PGL-2 with 30% excess Li, PGL-3 with 40% excess Li, and PGL-4 with 50% excess Li

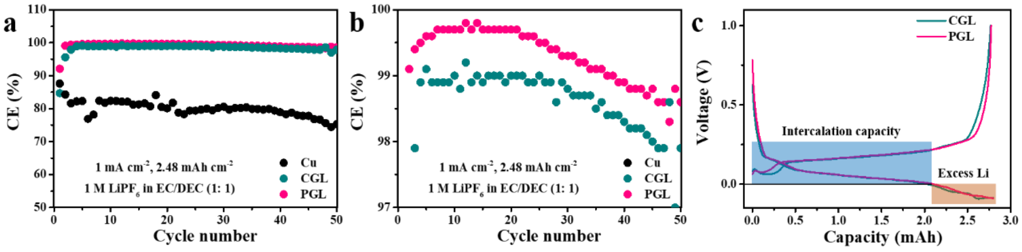

Fig. S7 a CE comparisons and **b** magnified CE values upon repeated cycling of bare Cu, CGL, and PGL at 1 mA cm^{-2} with a capacity of 2.48 mAh cm^{-2} in the traditional carbonate electrolyte consisting of 1 M $LiPF_6$ in EC/DEC (volume ratio of 1: 1). **c** The voltage profiles of CGL and PGL in the traditional carbonate electrolyte

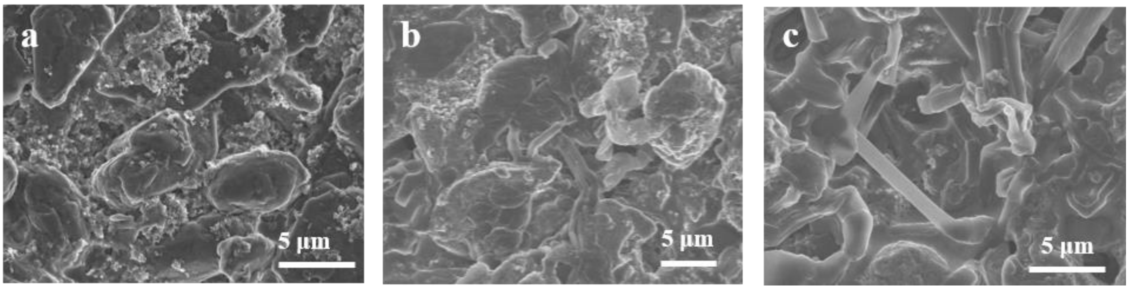

Fig. S8 Magnified top-view SEM images of CGL after **a** plating 10% excess Li, **b** plating 20% excess Li, and **c** plating 30% excess Li

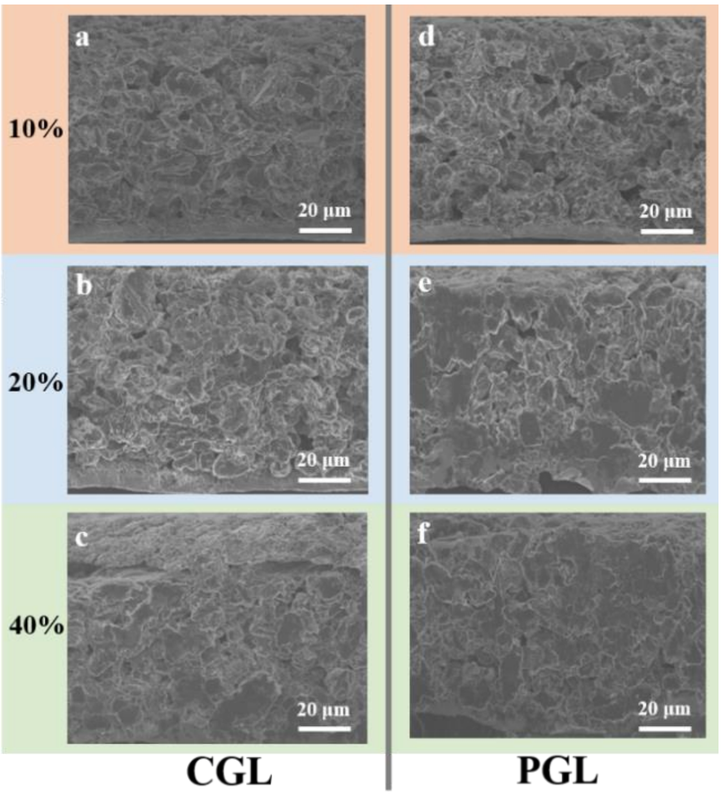

Fig. S9 Cross-sectional SEM images of CGL after **a** plating 10% excess Li, **b** plating 20% excess Li, **c** plating 40% excess Li. Cross-sectional SEM images of PGL after **d** plating 10% excess Li, **e** plating 20% excess Li, **f** plating 40% excess Li

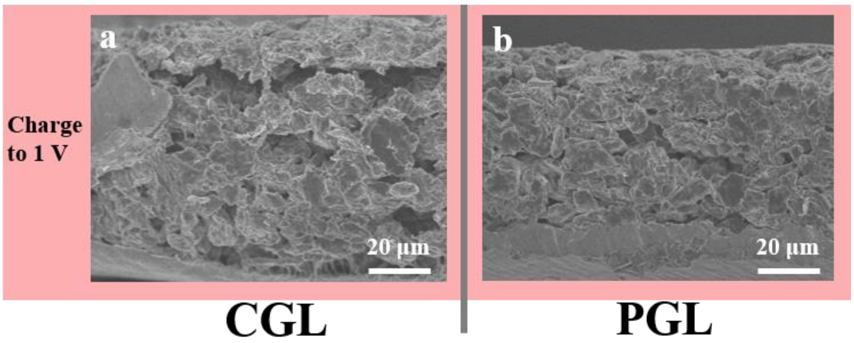

Fig. S10 Cross-sectional SEM images of **a** CGL and **b** PGL after charging to 1 V

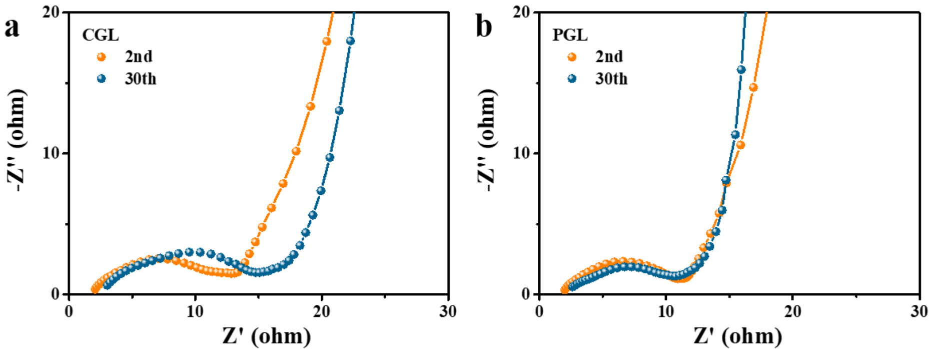

Fig. S11 EIS spectra of **a** CGL and **b** PGL electrodes at different cycles at 1 mA cm^{-2} when plating with 30% excess Li

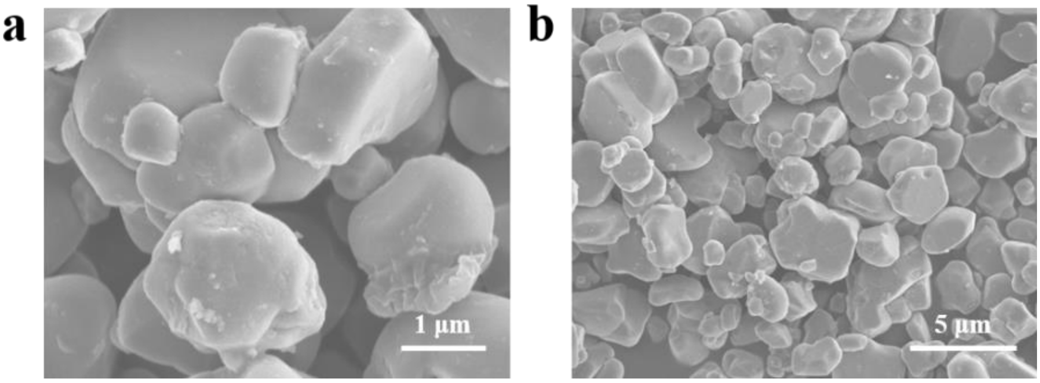

Fig. S12 SEM images of $LiNi_{0.8}Co_{0.1}Mn_{0.1}O_2$ (NCM) cathode material

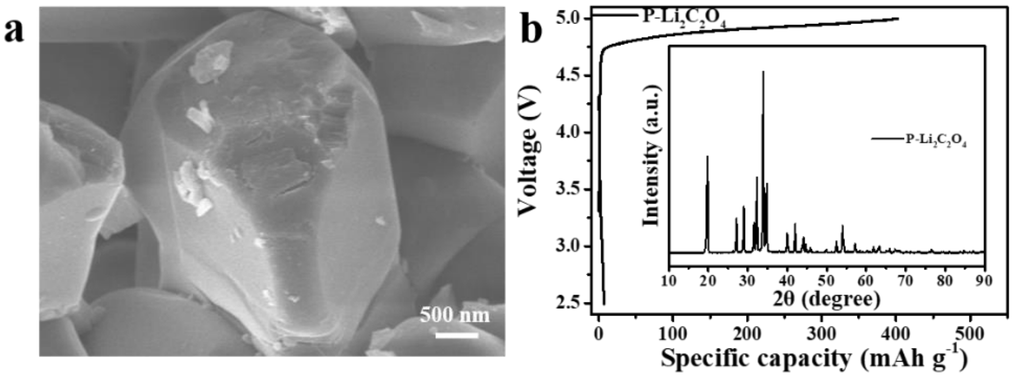

Fig. S13 a SEM image of pristine $Li_2C_2O_4$ (P-$Li_2C_2O_4$). **b** The charge/discharge curves of P-$Li_2C_2O_4$ at 500 mA g^{-1} (inset: XRD pattern)

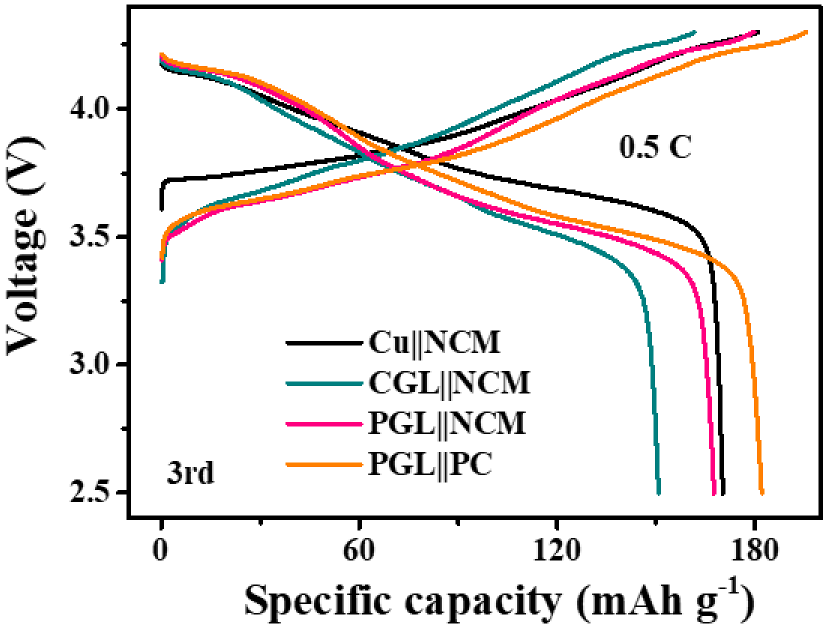

Fig. S14 The charge/discharge profiles for the third cycle of four kinds of full cells at 0.5 C between 2.5 V and 4.3 V

Table S1 Comprehensive comparison of anode fabrication, initial excess Li amount, employed electrolyte, anode reversibility, cathode composition, and full cell performance for representative reported Li metal batteries with initial Li-free anode or Li-less metal anode and this work

Anode	Fabrication	Initial excess Li	Electrolyte	CE of half cell	Cathode	Full cell performance	Refs.
Cu		0	0.6 M LiDFOB+0.6 M $LiBF_4$, FEC/DEC (1:2)	98.5% (1.5 mAh cm^{-2})	NCM523	90 cycles with 80% (2.4 mAh cm^{-2})	[S1]
Cu		0	4 M LiFSI, DME	99%	LFP	50 cycles with 60% (1.6 mAh cm^{-2})	[S2]
Cu@ SiO_x	Multiple lasing	0	4 M LiFSI, DME	99.3% (2 mAh cm^{-2})	LFP	100 cycles with 52.8% (2.6 mAh cm^{-2})	[S3]
Cu@ Ga/In/Sn	Dropping liquid metal	0	6 M LiFSI, DME	99.24% (5 mAh cm^{-2})	NCM811	50 cycles with 84% (4.5 mAh cm^{-2})	[S4]
Cu		0	6 M LiFSI, DME		Li-rich NCM811	100 cycles with 84% (5 mAh cm^{-2})	[S5]
Cu@ PVDF/LiF	Ball-milling and slurry coating	0	1 M LiTFSI, DOL/DME, 2% $LiNO_3$	96.6% (0.5 mAh cm^{-2})	LFP	80 cycles with 43% (0.8 mAh cm^{-2})	[S6]
Cu@ graphite	Slurry-coating	0	1.4 M LiFSI, DMC/ EC/BTFE (25:1:40)	99.5% (2.2 mAh cm^{-2})	NCM523	500 cycles with 80.2% (1.85 mAh cm^{-2})	[S7]
Cu@ graphite/Li	Slurry-coating and rolling with ultrathin Li foils	3.3	1.0 M LiFSI, DMC/HFE (1.8:2.0)		NCM523	210 cycles with 80% (3.0 mAh cm^{-2})	[S8]
Cu@ porous graphite lamina	Annealing, slurry-coating, template removing and heat reduction	1.3 (Electrochemical deposition)	1 M $LiPF_6$, EC/DEC, 10% FEC	98.6% (1 mAh cm^{-2})	NCM811	150 cycles with 80% (2.9 mAh cm^{-2})	[S9]
Cu@ graphite	Slurry-coating	0	1 M LiDFOB+0.4 M $LiBF_4$, FEC/DEC (1:2)	99.54%	NCM523	160 cycles with 80% (2.8 mAh cm^{-2})	[S10]
Cu		0	1.0 M LiTFSI+1.5 M LiFSI, G3, 10% HFE	99.3% (2 mAh cm^{-2})	NCM811 @Li_2O	300 cycles with 90% (4.85 mAh cm^{-2})	[S11]
**Cu@ PGL**	**Slurry-coating and template removing spontaneously**	**0**	**1 M $LiPF_6$, FEC/EMC/DEC (1:1:1)**	**99.5% (2.48 mAh cm^{-2})**	**NCM811@R-$Li_2C_2O_4$**	**300 cycles with 67.2% (2.65 mAh cm^{-2})**	**This work**

S2 Discussion

Table S1 shows the comparison of this work and recently reported representative Li metal batteries with initial Li-free anode or Li-less metal anode considering anode material, electrolyte component, cathode composition, and cell performance.

Firstly, the fabrication process of our PGL is scalable compared with other complex or

expensive methods (such as lasing and using Li foil or liquid metal) and could be integrated into existing battery manufacturing techniques.

Secondly, the reversibility of PGL can be greatly enhanced to 99.5% under a practical capacity depth of 2.48 mAh cm^{-2} in the commercialized high-voltage carbonate-based electrolyte without utilizing high concentration electrolyte with high viscosity or high-priced Li salt or fluorinated solvent. And the ultrahigh CE of 99.5% could satisfy the actual CE demand of Li-free metal anodes.

Thirdly, we report recrystallized $Li_2C_2O_4$ (R-$Li_2C_2O_4$) with high charge specific capacity (514.3 mAh g^{-1}), low reversibility (<10%), and moderate working voltage (4.7-5.0 V) as the cathode prelithiation reagent, which could be directly added to the cathode and is able to in-situ provide extra Li source for the Li-free metal anode. This strategy could avoid the handling of Li foil or electrochemical deposited Li metal, which could reduce the cost and complex procedures in fabricating composite Li anode. In addition, compared with the current reported Li_2O sacrificial salt with high reactivity in air, the R-$Li_2C_2O_4$ possesses excellent air-stability and the recrystallization process is feasible with low-cost, which significantly enhances the practicality of this cell configuration.

Finally, the assembled hybrid Li-ion/metal full cell utilizing prelithiated cathode and Li-free PGL anode delivers impressive capacity retention of 75% for 200 cycles and 67.2% for extended 300 cycles with improved CE values under high current (1.325 mA cm^{-2}) with high capacity cathode (2.65 mAh cm^{-2}), which outperforms the reported Li metal full cell with initial Li-free anode using carbonate-based electrolyte.

Therefore, based on the aforementioned discussion, the commercial feasibility of this high energy density hybrid Li-ion/metal full cell is demonstrated by these scalable approaches and the coordinated strategy of constructing high reversible symbiotic anode and introducing air-stable sacrificial cathode agent.

Supplementary References

[S1] R. Weber, M. Genovese, A.J. Louli, S. Hames, C. Martin et al., Long cycle life and dendrite-free lithium morphology in anode-free lithium pouch cells enabled by a dual-salt liquid electrolyte. Nat. Energy **4**, 683-689 (2019). https://doi.org/10.1038/s41560-019-0428-9

[S2] J. Qian, B.D. Adams, J. Zheng, W. Xu, W.A. Henderson et al., Anode-free rechargeable lithium metal batteries. Adv. Funct. Mater. **26**(39), 7094-7102 (2016). https://doi.org/10.1002/adfm.201602353

[S3] W. Chen, R.V. Salvatierra, M. Ren, J. Chen, M.G. Stanford et al., Laser-induced silicon oxide for anode-free lithium metal batteries. Adv. Mater. **32**(33), 2002850 (2020). https://doi.org/10.1002/adma.202002850

[S4] L. Lin, L. Suo, Y.S. Hu, H. Li, X. Huang et al., Epitaxial induced plating current-collector lasting lifespan of anode-free lithium metal battery. Adv. Energy Mater. **11**(9), 2003709 (2021). https://doi.org/10.1002/aenm.202003709

[S5] L. Lin, K. Qin, Q. Zhang, L. Gu, L. Suo et al., Li-rich $Li_2[Ni_{0.8}Co_{0.1}Mn_{0.1}]O_2$ for anode-free lithium metal batteries. Angew. Chem. Int. Ed. **60**(15), 8289-8296 (2021). https://doi.org/10.1002/anie.202017063

[S6] O. Tamwattana, H. Park, J. Kim, I. Hwang, G. Yoon et al., High-dielectric polymer coating for uniform lithium deposition in anode-free lithium batteries. ACS Energy Lett. **6**(12), 4416-4425 (2021). https://doi.org/10.1021/acsenergylett.1c02224

[S7] W. Cai, C. Yan, Y.X. Yao, L. Xu, X.R. Chen et al., The boundary of lithium plating in graphite electrode for safe lithium-ion batteries. Angew. Chem. Int. Ed. **60**(23), 13007-13012 (2021). https://doi.org/10.1002/anie.202102593

[S8] P. Shi, L.P. Hou, C.B. Jin, Y. Xiao, Y.X. Yao et al., A successive conversion-deintercalation delithiation mechanism for practical composite lithium anodes. J. Am. Chem. Soc. **144**(1), 212-218 (2022). https://doi.org/10.1021/jacs.1c08606

[S9] Y. Liu, X. Qin, F. Liu, B. Huang, S. Zhang et al., Basal nanosuit of graphite for high-energy hybrid Li batteries. ACS Nano **14**(2), 1837-1845 (2020). https://doi.org/10.1021/acsnano.9b07706

[S10] C. Martin, M. Genovese, A.J. Louli, R. Weber, J.R. Dahn, Cycling lithium metal on graphite to form hybrid lithium-ion/lithium metal cells. Joule **4**, 1296-1310 (2020). https://doi.org/10.1016/j.joule.2020.04.003

[S11] Y. Qiao, H. Yang, Z. Chang, H. Deng, X. Li et al., A high-energy-density and long-life initial-anode-free lithium battery enabled by a Li_2O sacrificial agent. Nat. Energy **6**, 653-662 (2021). https://doi.org/10.1038/s41560-021-00839-0
